# Hæmatinuria vs. Malarial Hæmaturia

**Published:** 1891-04

**Authors:** F. E. Young

**Affiliations:** Brownwood, Texas


For Daniel's Texas Medical Journal.

### HÆMATINURIA VS. MALARIAL HÆMATURIA.

BY F. E. YOUNG, M. D., BROWNWOOD, TEXAS.

IS THERE such a disease as malarial hæmaturia? Is not this nomenclature liable to mislead one not acquainted with this so-called disease (malarial hæmaturia) which I think is entirely symptomatic? Literally haematuria means, voiding blood by urine. This so-called malarial haematuria is not a true haemorrhage in any sense, but merely a pseudo-haemorrhage. From clinical observation and the pathology of the disease of which this is a sympton, (malarial poison) I think that haematinuria would be a more correct name and not so misleading, as it is the haematin thrown off with the urine from disintegation of the red blood corpuscles caused from malarial poison.

Some writers seem to think there is an additional cause, as they speak of there being more some special years than others. If *those* writers would stop and consider a moment, this would no doubt recall to mind the fact that those special years were excessively wet years, and hence a greater amount of malaria. Other writers also speak of sex and complexion being of more or less importance, as they claim that males are more prone to suffer from this so-called disease than females, as are likewise brunettes than blondes. My experience, to a certain extent, has proven the reverse, as I have treated a larger number of blondes than brunettes. The difference in experience of different observers, with regard to sex and complexion, is likely a coincidence.

### SYMPTOMS AND COURSE.

The condition resembles, in a great many respects, an ordinary case of pernicious fever, and in fact, is produced by the same cause, malarial poison. The patient has a cadaverous appearance owing to the loss of the coloring matter of the blood, which is often mistaken for icterus, or, as some term it, "extreme bilious condition." This death-like look and so-called haemorrhage comes on soon after the malarial paroxysm. There is general restlessness and sighing, caused by the breaking down of the red blood corpuscles, thus destroying their function as oxygen carriers, as well as shock to the nervous system from the same cause. The countenance is anxious; this is caused from fright, very often on account of its past fatality. The symptoms are so pathognomonic it is at once recognized by both patient and friends wherever it is indigenous. The tongue is either pale or furred or natural. Sometimes there is great thirst, with a sense of internal heat; the stomach is excessively irritable, and vomiting very common of mucus or muco-serous, or even a chocolate looking substance; then again having a bluish-green appearance, caused by decomposed secretions of the stomach, which is often mistaken for bile; the respirations are often irregular, and interrupted with a sense of oppression; restlessness is usual, but the mental faculties generally remain clear. The urine is of a dark color, and resembles blood, owing to the presence of haematin. Neither fibrin nor corpuscles are present. Albumen is often found, as in other cases of pernicious fever.

### TREATMENT:

There is but one remedy worth naming, and that is quinine. All others that have been tried have proven worthless, and are liable to injure the patient. The condition under consideration is produced by malarial poison. All observers agree on this point. This being the case, is it not plain that quinine, being almost a specific for all morbid conditions produced by malarial poison, would be the only remedy in this case? I take the same position as my friend, the late Austin Flint, M. D. In speaking of the treatment of malarial fever and their morbid conditions, he says: "It is always desirable to arrest the disease as speedily as possible; its morbid effects are less in proportion as it is quickly arrested, and the liability to relapse diminished." There is no need of preparatory treatment; begin with quinine at once, and give from 40 to 60 grains within the next ten hours following the paroxysm; any more would not likely be absorbed. Never use haemostatics of any kind. Morphine should never be used under any consideration, as it arrests the secretions sooner than any other agent that we can use, by its direct effect on the pareuchymatous structure of all the viscera, more especially the malpighian bodies of the kidneys; they become dammed up with the sediment of the urine which causes them to "strike work," thus converting a simple trouble, into an incurable one, viz: uremia. I have always resorted to potassii bromidum and chloral hydrate to quiet the restlessness. When the nausea is too great to give it by mouth, I give it per rectum, and also feed the patient the same way. Alcoholic stimulants should be used, and the diet should be of the most concentrated nourishment, such as soft boiled eggs, milk, etc. After the acute symptoms pass off, and the urine clears up, it is well to begin with ferruginous tonics and flesh producing agents.

